# Assessment of health facility quality improvements, United Republic of Tanzania

**DOI:** 10.2471/BLT.20.258145

**Published:** 2020-10-05

**Authors:** Anna D Gage, Talhiya Yahya, Margaret E Kruk, Eliudi Eliakimu, Mohamed Mohamed, Donat Shamba, Sanam Roder-DeWan

**Affiliations:** a Harvard T.H. Chan School of Public Health, 11th floor, Building 1, 665 Huntington Avenue, Boston, Massachusetts 02115, United States of America.; b Ministry of Health, Community Development, Gender, Elderly and Children, Dodoma, United Republic of Tanzania.; c East Central and Southern Africa Health Community, Arusha, United Republic of Tanzania.; d Ifakara Health Institute, Dar es Salaam, United Republic of Tanzania.

## Abstract

**Objective:**

To identify contextual factors associated with quality improvements in primary health-care facilities in the United Republic of Tanzania between two star rating assessments, focusing on local district administration and proximity to other facilities.

**Methods:**

Facilities underwent star rating assessments in 2015 and between 2017 and 2018; quality was rated from zero to five stars. The consolidated framework for implementation research, adapted to a low-income context, was used to identify variables associated with star rating improvements between assessments. Facility data were obtained from several secondary sources. The proportion of the variance in facility improvement observed at facility and district levels and the influence of nearby facilities and district administration were estimated using multilevel regression models and a hierarchical spatial autoregressive model, respectively.

**Findings:**

Star ratings improved at 4028 of 5595 (72%) primary care facilities. Factors associated with improvement included: (i) star rating in 2015; (ii) facility type (e.g. hospital) and ownership (e.g. public); (iii) participation in, or eligibility for, a results-based financing programme; (iv) local population density; and (v) distance from a major road. Overall, 20% of the variance in facility improvement was associated with district administration. Geographical clustering indicated that improvement at a facility was also associated with improvements at nearby facilities.

**Conclusion:**

Although the majority of facilities improved their star rating, there were substantial variations between facilities. Both district administration and proximity to high-performing facilities influenced improvements. Quality improvement interventions should take advantage of factors operating above the facility level, such as peer learning and peer pressure.

## Résumé

**Objectif:**

Identifier les facteurs contextuels liés aux améliorations de qualité dans les établissements de soins de santé primaires en République-Unie de Tanzanie, entre deux scores, en se concentrant sur l'administration locale et la proximité vis-à-vis d'autres établissements.

**Méthodes:**

Les établissements se sont vus attribuer un score de zéro à cinq étoiles en 2015 ainsi qu'un autre entre 2017 et 2018, afin d'évaluer leur qualité. C'est le cadre consolidé pour la recherche sur la mise en œuvre, adapté à ce pays à faible revenu, qui a été utilisé pour dégager des variables associées aux améliorations de score entre chaque période d'évaluation. Les données sur les établissements ont été collectées auprès de plusieurs sources secondaires. La proportion de la variance d'amélioration observée à l'échelle de l'établissement et du district ainsi que l'influence des établissements à proximité et de l'administration locale ont été mesurées à l'aide de modèles de régression multiniveaux pour l'un, et d'un modèle autorégressif spatial hiérarchique de l'autre.

**Résultats:**

Le score a augmenté dans 4028 des 5595 (72%) établissements de soins de santé primaires. Parmi les facteurs liés à cette amélioration, citons: (i) le nombre d'étoiles attribuées en 2015; (ii) le type d'établissement (un hôpital par exemple) et son propriétaire (l'État par exemple); (iii) la participation à ou l'éligibilité à un programme de financement fondé sur les résultats; (iv) la densité de population locale; et enfin, (v) la distance par rapport à un axe routier majeur. Au total, 20% de la variance d'amélioration de l'établissement était inhérente à l'administration du district. Et d'après le regroupement géographique, l'amélioration d'un établissement était également tributaire de celle des établissements à proximité.

**Conclusion:**

Bien que la majorité des établissements aient amélioré leur score, des écarts considérables subsistent entre eux. Tant l'administration de district que la proximité d'établissements hautement performants ont eu un impact sur leur progression. Les interventions visant à renforcer la qualité devraient tirer profit des facteurs situés hors des murs de l'établissement, tels que l'enseignement mutuel et la pression exercée par les pairs.

## Resumen

**Objetivo:**

Identificar los factores contextuales de las mejoras de la calidad en los establecimientos de atención primaria de salud de la República Unida de Tanzania entre las dos evaluaciones de clasificación por estrellas, en las que se presta especial atención a la administración local de los distritos y a la proximidad de otros establecimientos.

**Métodos:**

Se realizaron evaluaciones de clasificación por estrellas a los establecimientos en 2015 y entre 2017 y 2018; la calidad se clasificó de cero a cinco estrellas. El marco consolidado de la investigación sobre la aplicación, que se adaptó a un contexto de bajos ingresos, se utilizó para identificar las variables relacionadas con las mejoras en la clasificación por estrellas entre las evaluaciones. Los datos de los establecimientos se obtuvieron de varias fuentes secundarias. El porcentaje de la varianza en la mejora de los establecimientos que se observó a nivel de establecimiento y de distrito y la influencia de los establecimientos cercanos y de la administración del distrito se estimaron por medio de los modelos de regresión de multinivel y de un modelo autorregresivo espacial jerárquico, respectivamente.

**Resultados:**

La clasificación por estrellas mejoró en 4028 de 5595 (72 %) establecimientos de atención primaria. Los factores de la mejora incluyeron: i) la clasificación por estrellas de 2015; ii) el tipo de establecimiento (por ejemplo, hospital) y la propiedad (por ejemplo, pública); iii) la participación en un programa de financiamiento por resultados o la posibilidad de obtenerlo; iv) la densidad de población local; y v) la distancia a una carretera principal. En general, el 20 % de la variación en la mejora de los establecimientos se asoció a la administración del distrito. La agrupación geográfica indicó que la mejora de un establecimiento también estaba asociada con las mejoras de los establecimientos cercanos.

**Conclusión:**

Aunque la mayoría de los establecimientos mejoraron su clasificación por estrellas, se registraron variaciones significativas entre los establecimientos. Tanto la administración del distrito como la proximidad de los establecimientos de gran rendimiento influyeron en las mejoras. Las intervenciones de la mejora de la calidad deben aprovechar los factores que funcionan por encima del nivel del establecimiento, como el aprendizaje entre iguales y la presión social.

## ملخص

الغرض تحديد العوامل السياقية المرتبطة بتحسينات الجودة في مرافق الرعاية الصحية الأولية في جمهورية تنزانيا المتحدة بين تقييمين من تصنيفات النجوم، مع التركيز على إدارة المنطقة المحلية والقرب من المرافق الأخرى.

الطريقة خضعت المرافق لتقييمات تصنيف النجوم في عام 2015، وبين عامي 2017 و2018؛ وتم تصنيف الجودة من صفر إلى خمسة نجوم. إن الإطار الموحد لأبحاث التنفيذ، والذي تم تطويعه للتلاؤم مع أوضاع الدخل المنخفض، تم استخدامه لتحديد المتغيرات المرتبطة بتحسينات التصنيف باستخدام النجوم بين التقييمات. تم الحصول على بيانات المرافق من العديد من المصادر الثانوية. إن نسبة التنوع في تحسين المرفق، والذي لوحظ على المرفق ومستويات المناطق وتأثير المرافق القريبة وإدارة المنطقة، تم تقديرها باستخدام نماذج تحوف متعددة المستويات، ونموذج تحوفي تلقائي مكاني هيكلي، على الترتيب.

النتائج تحسنت تصنيفات النجوم في 4028 من 5595 (72%) من مرافق الرعاية الأولية. وشملت العوامل المرتبطة بالتحسين: (1) تصنيف النجوم في عام 2015؛ و(2) نوع المرفق (مستشفى مثلاً) والملكية (عامة مثلاً)؛ و(3) المشاركة في برنامج للتمويل يستند على النتائج، أو التأهل لمثل هذا البرنامج؛ و(4) كثافة السكان المحليين؛ و(5) المسافة من الطريق الرئيسي. بشكل عام، ارتبط 20% من التنوع في تحسين المرافق بإدارة المنطقة. أشار التجميع الجغرافي إلى أن التحسين في المنشأة كان قد ارتبط أيضًا بالتحسينات في المرافق القريبة.

الاستنتاج على الرغم من أن غالبية المرافق قد حسنت تصنيفها بالنجوم، إلا أنه كانت هناك اختلافات ملموسة بين المرافق. أثرت كل من إدارة المنطقة ومدى القرب من المرافق عالية الأداء، على التحسينات. يجب أن تستفيد تدخلات تحسين الجودة من العوامل التي تعمل فوق مستوى المرافق، مثل التعلم من الأقران وضغط الأقران.

## 摘要

**目的:**

在对坦桑尼亚联合共和国初级卫生保健机构进行两项星级评估时，确定与改进质量相关的背景因素，重点关注当地的地区行政管理和与其他机构的接近程度。

**方法:**

在 2015 年和 2017 年至 2018 年间参与星级评估的机构；质量被评为零到五星级。我们采用了针对低收入背景做相应调整后的综合实施研究框架，来确定两次评估之间与星级评估相关的变量。机构数据来源于多个次级资源。分别采用多级回归模型和层级空间自回归模型评估了在机构和地区层面观察的机构改进变化比例和附近机构和地区行政管理的影响。

**结果:**

5595 个初级护理机构中，4028 个机构的评估星级均有所提高 (72%)。与质量改进有关的因素包括：(i) 2015 年的评估星级；(ii) 机构类型（如医院）和所有权（如公立）；(iii) 对基于结果的融资项目的参与或参与资格；(iv) 当地人口密度以及 (v) 与主干公路的距离。总体来说，20% 的机构改进变化与地区行政管理有关。地理集群说明，单个机构的改进也与附近机构的改进有关。

**结论:**

尽管大多数机构的评估星级均有所提高，但不同机构之间仍存在重大差距。地区行政管理和与高效率机构的邻近度会影响改进效果。质量改进干预应充分利用超出机构层面运作的因素，如同行学习和同行压力。

## Резюме

**Цель:**

Определить контекстуальные факторы, связанные с совершенствованием качества работы учреждений первичной медико-санитарной помощи в Объединенной Республике Танзания, между двумя оценками по звездному рейтингу, уделяющему особое внимание местной районной администрации и близости к другим учреждениям.

**Методы:**

В 2015 году и в период с 2017 по 2018 год учреждения проходили оценку по звездному рейтингу. Качество оценивалось от нуля до пяти звезд. Для определения переменных, связанных с совершенствованием звездного рейтинга, между оценками использовалась консолидированная база для внедренческих исследований, адаптированная к контексту с низким уровнем доходов. Данные по учреждениям были получены из нескольких вторичных источников. Доля разницы в степени совершенствования качества работы учреждений, наблюдаемая на уровне учреждения и района, а также влияние близлежащих объектов и районной администрации прогнозировались с использованием многоуровневых регрессионных моделей и иерархической пространственной авторегрессионной модели соответственно.

**Результаты:**

Звездные рейтинги улучшились в 4028 учреждениях первичной медико-санитарной помощи из 5595 (72%). Связанные с улучшением факторы включали следующие: (i) звездный рейтинг в 2015 г.; (ii) тип учреждения (например, больница) и форма собственности (например, общественная); (iii) участие в программе финансирования, ориентированной на результаты, или право на участие в ней; (iv) плотность местного населения; (v) расстояние от главной дороги. В целом 20% разницы в степени совершенствования учреждений было связано с районной администрацией. Географическая разбивка показала, что совершенствование качества работы учреждения было также связано с улучшениями на близлежащих объектах.

**Вывод:**

Несмотря на то что большинство учреждений улучшили свой звездный рейтинг, наблюдалась существенная разница между учреждениями. На степень совершенствования влияла районная администрация и близость к высокопрофессиональным учреждениям. Мероприятия по повышению качества работы учреждений должны учитывать факторы, действующие выше уровня учреждения, такие как коллегиальное обучение и давление со стороны коллег.

## Introduction

Following the Astana declaration in 2018, primary health care is again high on the global health agenda and many countries are renewing their commitments to strengthening primary care.^1^ However, poor care quality is often a limiting factor. In the United Republic of Tanzania in 2016, an estimated 45 000 deaths were due to poor care quality and many involved conditions that could be addressed in primary care.^2^ Traditionally, quality improvement in health care has focused on micro-level approaches that rely on changing the practices of individual workers or facilities.^3^^,^^4^ However, these strategies may have a limited impact in complex, adaptive, health systems. Macro-level and meso-level strategies that affect whole systems or geographical areas are needed to address the social, political, economic and organizational structures underlying poor care quality.^3^^,^^5^

In the United Republic of Tanzania, the Health Quality Assurance Division of the health ministry did a star rating assessment in 2015 as part of a government initiative to improve service delivery.^6^^–^^8^ A data feedback approach was adopted. First, assessment teams, which comprised two independent health workers and one member of the local district’s health management team, collected data on care quality from all primary care facilities.^8^ Each facility was given a rating of between zero and five stars.^9^ Then, health facility administrators developed a quality improvement plan tailored to tackling specific quality gaps.^8^ Ratings were also discussed with district and regional health management teams.^8^ Some, but not all, administrations took an active interest and supported facilities. For example, some used the assessment tool as a supportive supervision checklist or encouraged facilities to learn from one another. However, decisions on whether and how to improve quality were taken locally; there was no universal plan and no national incentives for improvement. Originally, the health ministry planned to close facilities with zero stars but too many facilities met that criterion. Facilities were reassessed between 2017 and 2018.

Although the star rating assessment was the country’s flagship strategy for improving the quality of primary care, it was implemented at a time when other health system changes may have influenced quality. First, a larger government initiative for improving service delivery prioritized decentralization.^6^ Fiscal responsibility was delegated to local districts in 2014 and, in 2018, decentralized further to frontline health facilities through direct facility financing.^10^^,^^11^ Second, in 2015 a results-based financing programme was implemented in public facilities in eight of the country’s 31 regions to address health-care quality and utilization.^12^ A facility needed more than zero stars at baseline assessment or reassessment to be eligible for results-based financing. Facilities in programme regions that did not meet this criterion initially received a starter fund of 10 million Tanzanian shillings (about 4500 United States dollars). Once enrolled in the results-based financing programme, facilities’ performance was evaluated using criteria that differed from the star rating assessment, though with some overlap. Third, three additional regions and two districts in a fourth region received starter funds to improve quality in zero-star-rated public facilities, independently of the results-based financing programme.

The aim of our study was to identify micro- and meso-level factors associated with quality improvements at health-care facilities between two star rating assessment rounds in order to characterize the context in which quality improvement plans could be most effective. In particular, we determined whether improvements were related across groups of facilities by assessing how they were influenced by local district administration or geographical proximity to other facilities. Better understanding of how quality improvements are affected by the context in which a facility functions will help countries similar to the United Republic of Tanzania develop more targeted and effective strategies for quality improvement.

## Methods

### Conceptual framework

We adapted the consolidated framework for implementation research to make it applicable to a low-income context and suitable for a nationwide assessment.^13^^,^^14^ Details are available from the data repository.^15^ First, a structural environment was added to the outer setting domain and the inner setting domain was limited to the constructs of: (i) structural characteristics; (ii) networks and communications; and (iii) culture. Second, the modified framework conceptualized two pathways through which the outer setting could influence quality improvement: (i) district council administration (i.e. location within a district); and (ii) geographical proximity to other facilities.^16^ Policies, management, supervision and funds are the responsibility of the local district council, which is the lowest level of government charged with health facility administration in the country. Urban administrations included town councils and municipalities and all rural administrations were district councils – we use the term district council to refer to both rural and urban administrations.^17^ A facility’s location and immediate surroundings may independently influence its ability to implement quality improvement plans in low-income settings where facilities are isolated because of poor communications and high transportation costs. For example, proximity to a high-performing facility may encourage peer learning.

### Study sample

The 2015 baseline star rating assessment covered 6993 primary health-care facilities (i.e. public and private dispensaries, health centres and primary-level hospitals) in mainland United Republic of Tanzania. The assessment excluded: (i) facilities in Pemba and Zanzibar; (ii) national, zonal and regional referral hospitals; and (iii) stand-alone clinics, such as maternity homes and dental clinics. Reassessment took place between 2017 and 2018 and covered 7289 facilities. Our study included all facilities with star ratings from the two assessment rounds. Our analysis excluded: (i) the Dar es Salaam region because baseline assessment data were unavailable; (ii) institutional facilities, such as prisons, military and police facilities, and those with an unknown management type (2% of facilities); and (iii) facilities without geographical coordinates. Coordinates were obtained from the United Republic of Tanzania’s 2019 master health facility database.

### Dependent variable

The primary dependent variable was the change in a facility’s star rating between baseline assessment and reassessment. Star rating assessments covered four domains and twelve subdomains, which were awarded different score weightings (Table 1); they included measures of both structural quality (e.g. medicines and equipment) and process quality (e.g. adherence to clinical guidelines and patients’ experience), as assessed through facility audits, record reviews and interviews with providers and clients.^9^ Dispensaries, health centres and primary-level hospitals each had their own assessment tools, which included additional items as the level increased.^9^ The overall score ranged from 0 to 100%. Stars were awarded according to the lowest domain score: 0 to 19%: no stars; 20 to 39%: one star; 40 to 59%: two stars; 60 to 79%: three stars; 80 to 89%: four stars; and 90 to 100%: five stars. The analysis was repeated using the change in overall score as a secondary dependent variable.

**Table 1:** Scoring system, star rating assessment of health facility quality, United Republic of Tanzania, 2015–2020

Assessment domain, subdomain	Score weighting, %	Examples of indicators
**Health facility management and staff performance**		
Legality and licensing	0	Valid licence observed
Health facility management	10	Staff attendance register observed as complete
Use of facility data for planning and service improvement	5	Health management information system observed to be up to date
Staff performance	5	Providers aware of performance targets when interviewed
**Service charter fulfilment and accountability**		
Social accountability	10	Records of meetings indicate community participation
Client satisfaction	5	Interviewed clients have high average satisfaction scores
Organization of services	5	Schedule for facility outreach observed
Handling of emergency cases and referral system	10	Transportation of last documented referral took less than 1 hour
**Safe facilities conducive to health**		
Health facility infrastructure	10	Privacy ensured in consultation areas
Infection prevention and control	10	All service areas observed to have running water and soap
**Quality of care**		
Clinical services	15	Review of three antenatal care records indicate adherence to clinical guidelines (e.g. iron supplementation)
Clinical support services	15	Essential medicines observed as available

### Independent variables

We identified contextual factors that could influence a facility’s ability to improve quality using the modified conceptual framework. Values for independent variables were obtained from a range of data sources (Table 2), preferably for 2015 to correspond with the time of baseline assessment. Variables obtained from Demographic and Health Surveys were calculated for individual districts and applied to all facilities in the district. As these surveys are representative only at the regional level and some districts had very small sample sizes, we smoothed the variations arising from the small sample sizes by calculating predicted values for the variables using a null, three-level, random intercept model with households nested within districts and regions.^23^

**Table 2:** Independent variables, assessment of changes in health facility quality, United Republic of Tanzania, 2015–2020

Construct and facility variable	Definition of variable	Data source	Mean (SD)^a^
**Outer setting**			
Patient needs and resources			
Population density	No. of people within a 5 km radius of a health facility^b^	World population estimates for 2015^18^	24 147 (63 733)
Population demand for coverage^c^	Percentage of women in district who gave birth in a facility in the past 5 years	Demographic and Health Survey 2016^19^	71 (22)
Informed consumers^c^	Percentage of women in district who completed primary education	Demographic and Health Survey 2016	73 (15)
Health-care agency^c^	Percentage of women in district who were involved in decisions about their own health care	Demographic and Health Survey 2016	74 (13)
Cosmopolitanism			
Facility density^c^	Number of facilities in district per 100 000 population	Star rating assessment 2015	15.4 (7.2)
Urban council^c^	Percentage of facilities in town or municipal council areas and not in rural district council areas	Star rating assessment 2015	12 (33)
Structural environment			
Accessibility	Distance to major road, in km (bilinear interpolation)^b^	OpenStreetMap 2016^20^	2.32 (4.54)
Remoteness	Distance to city with a population of at least 50 000, in 10-km units	Natural Earth II^21^	7.1 (5.1)
Peer pressure			
Facility rank at baseline	Percentile rank of facility’s baseline star rating compared with other facilities in the same district	Star rating assessment 2015	42 (33)
External policies and incentives			
Participated in results-based financing programme	Percentage of facilities that participated in the results-based financing programme	Star rating assessment 2015	15 (NA)
Ineligible for results-based financing programme	Percentage of facilities that were public facilities in a region participating in the results-based financing programme but had a baseline star rating of zero	Star rating assessment 2015	11 (NA)
Starter fund	Percentage of facilities in an area eligible for a starter fund that had a baseline star rating of zero	Star rating assessment 2015	4 (NA)
**Inner setting**			
Structural characteristics			
Ownership	Percentage of facilities that were public	Star rating assessment 2015	81 (NA)
Ownership	Percentage of facilities that were private for-profit facilities	Star rating assessment 2015	9 (NA)
Ownership	Percentage of facilities that were private non-profit facilities	Star rating assessment 2015	10 (NA)
Level	Percentage of facilities that were dispensaries	Star rating assessment 2015	85 (NA)
Level	Percentage of facilities that were health centres	Star rating assessment 2015	12 (NA)
Level	Percentage of facilities that were primary-level hospitals	Star rating assessment 2015	3 (NA)
Baseline performance	Facility star rating at baseline	Star rating assessment 2015	0.81 (0.71)
**Subsample analysis only^d^**			
External policies and incentives			
External supervision	Percentage of facilities visited by an external supervisor in the previous 6 months who used a checklist, discussed facility performance and helped the facility make decisions based on data	Service provision assessment^22^ 2014–2015	76 (NA)
Structural characteristics			
Human resources	Number of full-time health workers in each facility^b^	Service provision assessment 2014–2015	8.6 (21.9)
Culture			
Routine data use	Percentage of facilities that reported routine use of a quality assurance system	Service provision assessment 2014–2015	15 (NA)
Client responsiveness	Percentage of facilities with a procedure for reviewing patient feedback	Service provision assessment 2014–2015	9 (NA)
Community engagement	Percentage of facilities that had a staff–community meeting within the previous 6 months	Service provision assessment 2014–2015	64 (NA)
Management function	Percentage of facilities that acted after a recent management meeting	Service provision assessment 2014–2015	46 (NA)

NA: not applicable; SD: standard deviation.

^a^ All values are means and standard deviations unless otherwise noted.

^b^ The natural log of this variable was used in the analytical models.

^c^ Council-level variable.

^d^ The subsample analysis included 672 facilities that took part in a service provision assessment between 2014 and 2015.

As limited data were available on inner setting characteristics, we performed a secondary analysis on a subset of facilities covered by the service provision assessment carried out from 2014 to 2015 (Table 2).^22^ Service provision assessments are nationally representative facility surveys that include data on facility management and provider motivation. Facilities covered by this assessment were linked to star rating data using geographical coordinates. Data on other covariates came from WorldPop, OpenStreetMap and Natural Earth.^18^^,^^20^^,^^21^

### Analysis

Guided by the conceptual framework, we estimated the contribution of the covariates to quality improvement using a two-level, random intercept model with facilities nested within districts. The percentage variation in improvement explained by a set of covariates was calculated as the difference between the variance in the adjusted model (which included these covariates) and the null model (which did not include any covariates) divided by the null model variance. For a subsample of facilities, we repeated the calculations using a full random intercept model that included data on additional variables available only from the service provision assessment.

We examined the contribution of the geographical proximity of facilities to quality improvement using spatial analyses. First, we mapped the improvement using interpolation with an inverse distance weighting and clipped to 10 km around the facility to visualize trends. We calculated Moran’s *I* for the change in star rating between the two assessments. Moran’s *I* provides a measure of spatial autocorrelation by comparison with the null hypothesis of complete spatial randomness.^24^ An inverse distance weighting matrix (i.e. with a weighting of 1/*x^2^*, where *x* is the distance between facilities) was applied for facilities within 50 km of the index facility. Furthermore, the residuals of the two-level, random intercept model were also tested for spatial autocorrelation that was not explained by district or other covariates.

Finally, as we found that the residuals were still autocorrelated, we used a hierarchical spatial autoregressive model to explicitly model spatial relationships at both facility and district levels.^25^ This model included spatial lag terms at facility and district levels: these terms can be interpreted as associations between the improvement in a facility’s star rating and improvements in nearby facilities and in adjacent districts, respectively. Additional details of the hierarchical spatial model are available from the data repository.^15^

The National Institute for Medical Research of the United Republic of Tanzania and the Ifakara Health Institute Institutional Review Board approved the original study and the Harvard Institutional Review Board determined that this secondary analysis did not involve research on human subjects. All analyses were conducted in R v. 3.6.1 (The R Foundation, Vienna, Austria).

## Results

Star rating scores from two assessments were available for 5595 facilities that met inclusion criteria. Overall, 81% (4534/5595) were public facilities and 85% (4777/5595) were dispensaries (Table 2). Facility performance at baseline was poor: 34% (1927/5595) scored zero stars and 52% (2892/5595) scored one star. In total, 15% (835/5595) of facilities participated in the results-based financing programme and a further 11% (637/5595) were public facilities in programme regions that were ineligible because they had zero stars. There was an average of 47 facilities per district and 15 facilities per 100 000 people. Of 672 facilities with data available from the service provision assessment, 76% (508/672) had received external supervision in the past 6 months and 15% (99/672) had undergone routine quality assurance before star rating assessments started in 2015.

**Fig. 1:**
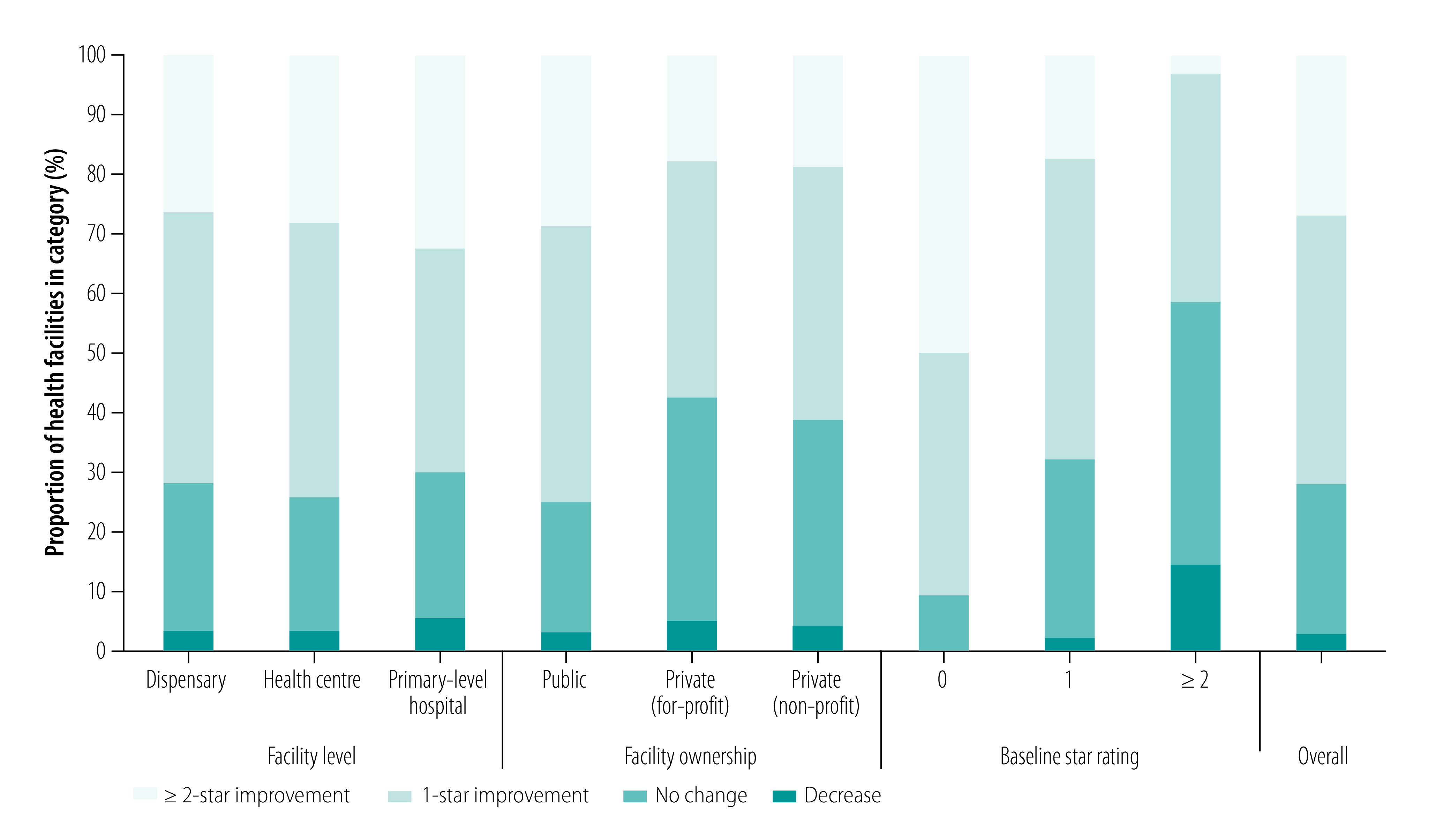
Improvement in star rating,^a^ by facility level, ownership and baseline star rating, 5595 health facilities, United Republic of Tanzania, 2015–2018

^a^ The improvement in star rating (a measure of care quality) was between the baseline assessment in 2015 and reassessment during 2017 and 2018.

Fig. 1 shows the proportion of facilities with a changed star rating between baseline and reassessment. Overall, 3% (181/5595) had a lower star rating at reassessment, 25% (1386/5595) received the same score, 45% (2531/5595) improved by one star and 27% (1497/5595) improved by two or more stars. There was no difference in improvements between dispensaries, health centres and primary-level hospitals. Public facilities improved more than for-profit and non-profit private facilities. There was a strong association with baseline rating: facilities with zero stars at baseline showed the largest improvements. Facilities with a lower score at reassessment than baseline more often had a score of two or higher at baseline and were more often a for-profit or non-profit private facility. Although scores decreased across all domains in facilities with lower reassessment scores, the largest declines were in the service charter fulfilment and accountability domain.

**Fig. 2:**
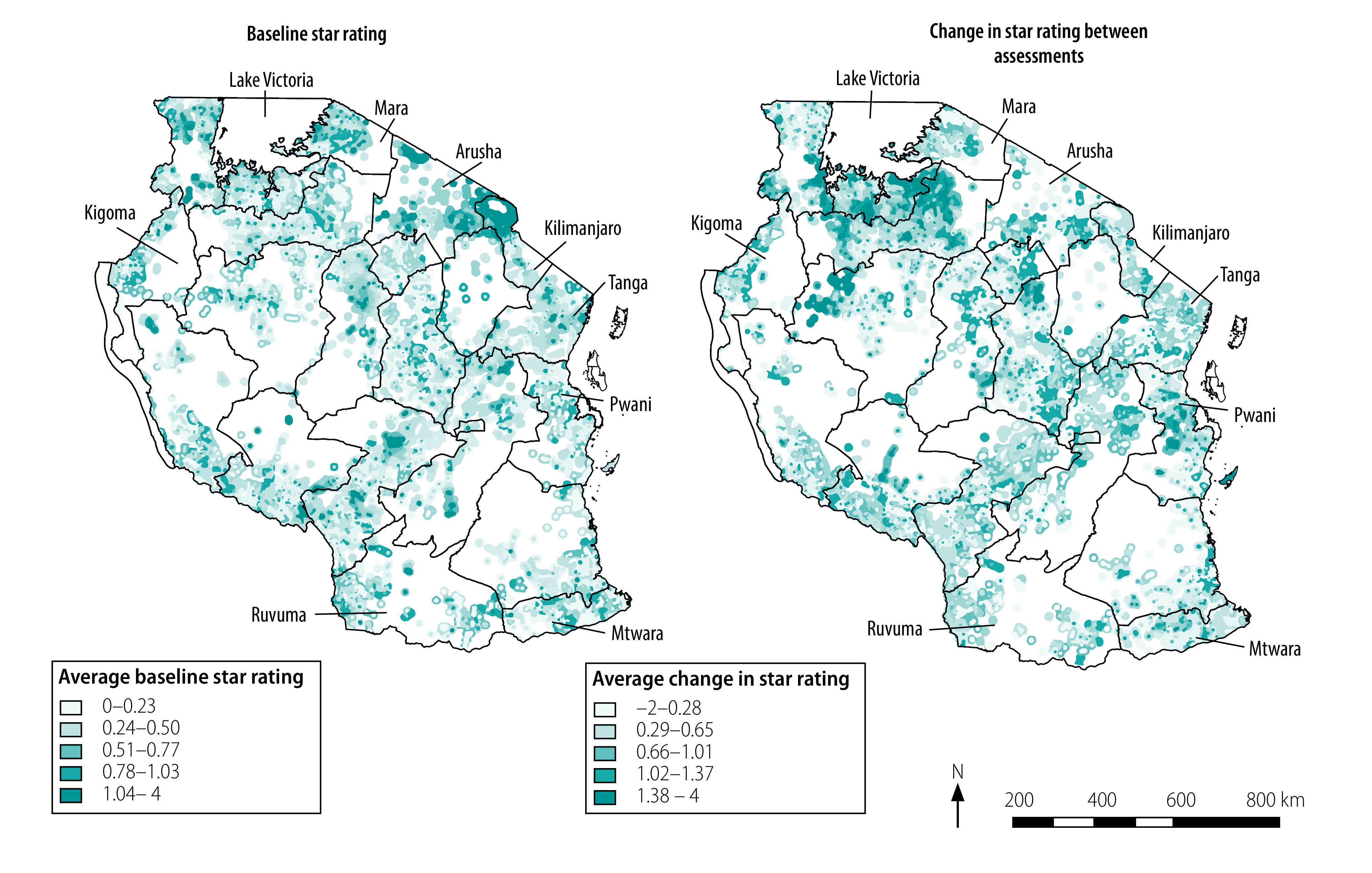
Baseline star rating and improvement in star rating,^a^ 5595 health facilities, United Republic of Tanzania, 2015–2018

^a^ The improvement in star rating (a measure of care quality) was between the baseline assessment in 2015 and reassessment during 2017 and 2018.

Source: National and regional boundaries are from GADM (Database of Global Administrative Areas) (https://gadm.org/).

Fig. 2 shows the baseline star rating and improvement in star rating for all 5595 facilities assessed. At baseline, star ratings were best in Arusha and Kilimanjaro regions and poorest in Kigoma and Mtwara regions. Facilities improved most in the Pwani region and in regions surrounding Lake Victoria, except the Mara region. Facilities improved least in the Mara, Tanga and Ruvuma regions. There was significant geographical clustering of both baseline ratings (Moran’s *I*: 0.17; *P* < 0.01) and star rating improvements (Moran’s *I*: 0.18; *P* < 0.01).

Table 3 shows the results of the random intercept models based on the full study sample of 5595 facilities. With the null model, 20% of the variance in facility improvement was due to the variance between districts, whereas 80% was due to the variance between facilities within districts (see footnotes to Table 3). With the outer setting model, 29% of the total variance was explained by all covariates included in the model. With the full model, which also included inner setting variables with data available for all facilities, 33% of the total variance was explained by the covariates. Relative to baseline performance, primary-level hospitals and health centres improved more than dispensaries, and public facilities improved more than for-profit and non-profit private facilities. In addition, higher population density around a facility and the facility’s proximity to a major road were significantly associated with greater improvements. Participation in the results-based financing programme was associated with an average 0.37-star improvement (95% confidence interval, CI: 0.27 to 0.46), whereas being ineligible for the programme was associated with a 0.60-star improvement (95% CI: 0.50 to 0.70). Receipt of a starter fund was not significantly associated with an improvement. For every one-star increment in a facility’s baseline score, there was a 0.69-star decrease (95% CI: −0.78 to −0.60) in performance, which indicates that initially low-performing facilities improved more than others, irrespective of other contextual factors.

**Table 3:** Variables associated with improvement in star rating,^a^ by analytical model, 5595 health facilities, United Republic of Tanzania, 2015–2018

Facility variable	Regression coefficient (95% CI),^b^ in star rating units				
	Two-level, random intercept model				HSAR model
	Null model	Outer setting model^c^	Full model^d^		
**No. of people within a 5 km radius of health facility^e^**	NA	0.07 (0.05 to 0.09)	0.07 (0.05 to 0.10)		0.07 (0.05 to 0.09)
**Proportion of women in district who gave birth in a facility in the past 5 years**	NA	0.20 (−0.22 to 0.62)	0.34 (−0.09 to 0.78)		0.30 (0.11 to 0.49)
**Proportion of women with primary education in district**	NA	0.04 (−0.56 to 0.63)	0.24 (−0.37 to 0.85)		0.23 (−0.06 to 0.51)
**Proportion of women involved in decisions about health care in district**	NA	−0.35 (−1.08 to 0.38)	−0.46 (−1.2 to 0.29)		NA
**No. of facilities per 100 000 population in district**	NA	0.00 (−0.01 to 0.01)	−0.01 (−0.02 to 0.00)		0.00 (−0.01 to 0.00)
**Located in urban district**	NA	−0.01 (−0.22 to 0.20)	−0.01 (−0.23 to 0.21)		0.06 (−0.04 to 0.16)
**Distance to major road^e^**	NA	−0.01 (−0.02 to 0.00)	−0.01 (−0.02 to 0.00)		−0.01 (−0.02 to 0.00)
**Distance to large city^f^**	NA	0.01 (0.00 to 0.01)	0.00 (0.00 to 0.01)		0.00 (−0.01 to 0.00)
**Percentile rank of facility’s baseline star rating among facilities in the same district**	NA	−1.14 (−1.20 to −1.07)	0.03 (−0.15 to 0.20)		−0.18 (−0.34 to −0.01)
**Participating in results-based financing programme**	NA	0.52 (0.44 to 0.61)	0.37 (0.27 to 0.46)		0.29 (0.20 to 0.38)
**Ineligible for results-based financing programme**	NA	0.65 (0.56 to 0.75)	0.60 (0.50 to 0.70)		0.50 (0.40 to 0.59)
**Receipt of a starter fund**	NA	0.09 (−0.03 to 0.20)	0.10 (−0.01 to 0.21)		0.08 (−0.03 to 0.19)
**Facility ownership (reference: public)**					
Private for-profit	NA	NA	−0.29 (−0.37 to −0.22)		−0.32 (−0.39 to −0.24)
Private non-profit	NA	NA	−0.10 (−0.16 to −0.03)		−0.11 (−0.17 to −0.04)
**Facility level (reference: dispensary)**					
Health centre	NA	NA	0.36 (0.31 to 0.42)		0.34 (0.28 to 0.40)
Primary-level hospital	NA	NA	0.76 (0.65 to 0.87)		0.74 (0.63 to 0.85)
**Baseline star rating**	NA	NA	−0.69 (−0.78 to −0.60)		−0.58 (−0.66 to −0.50)
**First-level lag term (facility)^g^**	NA	NA	NA		0.34 (0.27 to 0.40)
**Second-level lag term (district)^g^**	NA	NA	NA		0.36 (0.10 to 0.61)
**Constant**	1.01 (0.93 to 1.08)	0.75 (0.18 to 1.31)	0.72 (0.15 to 1.29)		0.31 (−0.01 to 0.62)
**Variance from districts^h,i,j,k^**	0.16	0.10	0.11		0.05
**Variance from facilities^h,i,j,k^**	0.63	0.46	0.42		0.44
**Moran's *I* for residuals (*P* value)**	0.05 (< 0.01)	0.04 (< 0.01)	0.05 (< 0.01)		NA

CI: confidence interval; HSAR: hierarchical spatial autoregressive; NA: not applicable.

^a^ The improvement in star rating (a measure of care quality) was between the baseline assessment in 2015 and reassessment during 2017 and 2018.

^b^ The table shows regression coefficients and 95% CIs, except where otherwise stated.

^c^ The outer setting model included outer setting variables listed in Table 2 with data available for all facilities.

^d^ The full model included inner and outer setting variables listed in Table 2 with data available for all facilities.

^e^ The natural log of this variable was used in the analytical models.

^f^ Distance was expressed in 10-km units.

^g^ Spatial lag terms at facility and district levels represent associations between improvement in a facility’s star rating and improvements in nearby facilities and adjacent districts, respectively.

^h^ With the null model, the proportion of the variance associated with districts was 20% (i.e. 0.16 / (0.16+0.63) × 100) and the proportion associated with facilities was 80% (i.e. 0.63 / (0.16+0.63) × 100).

^i^ With the outer setting model, the proportion of the total variance explained by all covariates included was 29% (i.e. {[(0.16+0.63)−(0.10+0.46)] / (0.16+0.63)} × 100).

^j^ With the full model, the proportion of the total variance explained by all covariates included was 33% (i.e. {[(0.16+0.63)−(0.11+0.42)] / (0.16+0.63)} × 100).

^k^ With the hierarchical spatial autoregressive model, the proportion of the total variance explained by all covariates included was 38% (i.e. {[(0.16+0.63)−(0.05+0.44)] / (0.16+0.63)} × 100).

The results of models based on the subsample of 672 facilities covered by the service provision assessment are presented in Table 4 (available at: http://www.who.int/bulletin/volumes/98/12/20-258145). With the null model, 18% of the variance in facility improvement was due to the variance between districts (see footnotes to Table 4). With the full model, which included all variables in the full model in Table 3, the covariates explained 39% of the total variance. With the final model, the addition of data on variables included in the service provision assessment contributed only one percentage point to the explained variance. However, this model identified two additional variables associated with a greater improvement in star rating: the number of full-time health workers in each facility and routine use of a quality assurance system. Models based on the change in overall score gave similar findings.^15^

**Table 4:** Variables associated with improvement in star rating,^a^ by analytical model, subsample of 672 health facilities,^b^ United Republic of Tanzania, 2015–2018

Facility variable	Regression coefficient (95% CI),^c^ in star rating units		
	Two-level, random intercept model		
	Null model	Full model^d^	Additional inner setting model^e^
**No. of people within a 5 km radius of health facility^f^**	NA	0.03 (−0.03 to 0.10)	0.00 (0.00 to 0.00)
**Proportion of women in district who gave birth in a facility in the past 5 years**	NA	0.29 (−0.25 to 0.83)	0.29 (−0.26 to 0.84)
**Proportion of women with primary education in district**	NA	0.23 (−0.49 to 0.95)	0.35 (−0.38 to 1.08)
**Proportion of women involved in decisions about health care in district**	NA	−0.69 (−1.58 to 0.20)	−0.72 (−1.62 to 0.19)
**No. of facilities per 100 000 population in district**	NA	−0.01 (−0.02 to 0.00)	−0.01 (−0.02 to 0.00)
**Located in urban district**	NA	0.21 (−0.07 to 0.49)	0.20 (−0.08 to 0.48)
**Distance to major road^f^**	NA	0.02 (0.00 to 0.05)	0.01 (0.00 to 0.02)
**Distance to large city^g^**	NA	0.00 (−0.01 to 0.02)	0.01 (−0.01 to 0.02)
**Percentile rank of facility’s baseline star rating among facilities in the same district**	NA	0.03 (−0.39 to 0.44)	−0.05 (−0.46 to 0.35)
**Participating in results-based financing programme**	NA	0.16 (−0.03 to 0.34)	0.17 (−0.01 to 0.36)
**Ineligible for results-based financing programme**	NA	0.51 (0.25 to 0.76)	0.49 (0.24 to 0.75)
**Receipt of a starter fund**	NA	−0.13 (−0.54 to 0.27)	−0.04 (−0.44 to 0.36)
**Facility ownership (reference: public)**			
Private for-profit	NA	−0.36 (−0.60 to −0.13)	−0.37 (−0.61 to −0.13)
Private non-profit	NA	−0.10 (−0.26 to 0.05)	−0.07 (−0.23 to 0.08)
**Facility level (reference: dispensary)**			
Health centre	NA	0.46 (0.34 to 0.58)	0.15 (−0.01 to 0.31)
Primary-level hospital	NA	0.86 (0.68 to 1.03)	0.19 (−0.12 to 0.49)
**Baseline star rating**	NA	−0.68 (−0.86 to −0.51)	−0.68 (−0.85 to −0.51)
**Additional inner setting variables^e^**			
Visited by an external supervisor in the previous 6 months	NA	NA	0.12 (−0.02 to 0.25)
Number of full-time health workers in each facility**^f^**	NA	NA	0.18 (0.10 to 0.26)
Reported routine use of a quality assurance system	NA	NA	0.19 (0.06 to 0.32)
Had a procedure for reviewing patient feedback	NA	NA	−0.04 (−0.16 to 0.08)
Had a staff–community meeting in the last 6 months	NA	NA	−0.06 (−0.21 to 0.09)
Acted after a recent management meeting	NA	NA	0.02 (−0.10 to 0.14)
**Constant**	1.01 (0.92 to 1.11)	1.37 (0.53 to 2.22)	1.31 (0.62 to 1.99)
**Variance from districts^h,i^**	0.14	0.07	0.08
**Variance from facilities^h,i^**	0.65	0.41	0.38

CI: confidence interval; NA: not applicable.

^a^ The improvement in star rating (a measure of care quality) was between the baseline assessment in 2015 and reassessment during 2017 and 2018.

^b^ The subsample of 672 facilities took part in a service provision assessment between 2014 and 2015.

^c^ The table shows regression coefficients and 95% CIs, except where otherwise stated.

^d^ The full model includes all variables in the full model in Table 3.

^e^ The additional inner setting model included variables from the 2014–2015 service provision assessment (Table 2) in addition to variables included in the full model.

^f^ The natural log of this variable was used in the analytical models.

^g^ Distance was expressed in 10-km units.

^h^ With the null model, the proportion of the variance associated with districts was 18% (i.e. 0.14 / (0.14+0.65) × 100).

^i^ With the full model, the proportion of the total variance explained by all covariates included was 39% (i.e. {[(0.14+0.65)−(0.07+0.41)] / (0.14+0.65)} × 100).

With the random intercept models, Moran’s *I* for the residuals was 0.05 (Table 3), significantly lower than the unadjusted value of 0.18 (*P* < 0.05), which indicates that a large portion of the spatial autocorrelation was due to the facilities’ district. However, the significant autocorrelation of the residuals suggests that spatial factors other than district may be associated with quality improvement. The results of the hierarchical spatial autoregressive model that included spatial lag terms at both levels are also presented in Table 3. Both lag terms were large and significantly associated with quality improvement. In this model, covariates accounted for 38% of the total variance (see footnote to Table 3). In addition, the proportion of women in a district who gave birth in a facility and the baseline rank of a facility relative to other facilities in the same district were also significantly associated with the improvement in star rating. Facilities with a low baseline rank showed greater improvements.

## Discussion

The success of quality improvement interventions in health facilities depends on processes within facilities and the context in which they operate. Our study in the United Republic of Tanzania found that both district and proximity to high-performing facilities influenced a facility’s ability to improve care quality, as assessed using the star rating system. The district accounted for approximately 20% of the variance in improvement.

In addition, baseline star rating, facility type and participation in, or ineligibility for, results-based financing were also important predictors of improvement. For example, facilities that were ineligible for results-based financing because of a low star rating improved more than facilities that received funding through results-based financing. The incentive of becoming eligible may have had a greater effect than incentives provided by results-based financing itself. Also, district councils may have put pressure on ineligible facilities to pursue additional funding. Primary-level hospitals and public facilities were more likely to improve than dispensaries or privately managed facilities. Private facilities may have felt less pressure to improve given their independent funding or received less support from district council administrations. Dispensaries may have had fewer financial and human resources to devote to improvement than primary-level hospitals.

More research is needed to understand the causal mechanisms behind our findings. Qualitative interviews with facility managers conducted by the Ifakara Health Institute confirmed the importance of context (Sanam Roder-DeWan, Ifakara Health Institute, unpublished observations, 2019). For example, managers noted that star ratings stimulated competition between neighbouring facilities, with some facilities being envious of surrounding facilities’ high baseline scores. Managers also thought district council administration was critical for clarifying and strengthening facilities’ accountability, both to the council administration and the community.

This study has several limitations. First, limited data were available, particularly for adapting the consolidated framework for implementation research to a national programme in a low-income setting.^13^ Ideally, it would be helpful to have more data on: (i) interactions between district councils and facilities; (ii) facilities’ culture and readiness to change; and (iii) health worker characteristics, such as self-efficacy and knowledge of the intervention. Supplementary data from the service provision assessment were still limited in these areas. Second, the star rating tool is limited in the way it assesses quality: (i) it does not consider health outcomes data; and (ii) it includes a large number of input variables, which may have overemphasized their importance relative to process variables. Moreover, data on users’ experiences may have been influenced by their varied and growing expectations of the health-care system.^26^ The health ministry is revising the star rating tool to put less emphasis on inputs in future. Finally, the collection of star rating data by health workers affiliated with district health management teams had two consequences: (i) we were unable to disentangle the variance due to district council administration from the variance due to data collectors; and (ii) we were unable to tell if some council health management teams had inflated their ratings during reassessment to show greater improvements.

Despite these limitations, our study has implications for policy. First, our findings suggest that the data feedback strategy did not operate simply at the micro level, where only facility-level characteristics influence quality improvements. Instead, meso- and macro-level factors, such as district council administration, peer learning and pressure from neighbouring facilities, were also important. Health system interventions that target districts could be explicitly designed to take advantage of the influence of health facilities and networks within those districts.^27^ In the next star rating assessment in the country, facilities will be given certificates to post publicly to promote social accountability and encourage peers. Second, the strong association we found between quality improvement and baseline performance indicates there was a strong floor effect, or regression to the mean: most improvements occurred in low-performing facilities. When many facilities have one or two stars, new strategies may be required to promote further improvement. Finally, the difference in improvement between facilities ineligible for, and enrolled in, a results-based financing programme indicates that incentives for such financing could be redesigned.^28^
